# VEGFR-2 Expression in Glioblastoma Multiforme Depends on Inflammatory Tumor Microenvironment

**DOI:** 10.1155/2015/385030

**Published:** 2015-12-22

**Authors:** Jana Jaal, Marju Kase, Ave Minajeva, Mikk Saretok, Aidi Adamson, Jelizaveta Junninen, Tõnis Metsaots, Tõnu Jõgi, Madis Joonsalu, Markus Vardja, Toomas Asser

**Affiliations:** ^1^Hematology and Oncology Clinic, Department of Radiotherapy and Oncological Therapy, Tartu University Hospital, 51003 Tartu, Estonia; ^2^Faculty of Medicine, University of Tartu, 50411 Tartu, Estonia; ^3^East-Tallinn Central Hospital, Center of Oncology, 10138 Tallinn, Estonia; ^4^Neurology Clinic, Department of Neurosurgery, Tartu University Hospital, 51014 Tartu, Estonia

## Abstract

Glioblastoma multiforme (GBM) is one of the most angiogenic tumors. However, antiangiogenic therapy has not shown significant clinical efficacy. The aim of our study was to evaluate the impact of inflammatory tumor microenvironment on the expression of vascular endothelial growth factor receptor 2 (VEGFR-2). Surgically excised primary GBM tissues were histologically examined for overall extent of inflammation (score 1–3). After immunohistochemistry, the tissue expression of ICAM-1 (optical density), the number of VEGFR-2 positive (VEGFR-2+) blood vessels (per microscopic field), and the endothelial staining intensity of VEGFR-2 (score 0–3) were determined. In GBM, the extent of inflammation was 1.9 ± 0.7 (group mean ± SD). Mean optical density of inflammatory mediator ICAM-1 was 57.0 ± 27.1 (pixel values). The number of VEGFR-2+ blood vessels and endothelial VEGFR-2 staining intensity were 6.2 ± 2.4 and 1.2 ± 0.8, respectively. A positive association was found between endothelial VEGFR-2 staining intensity and the extent of inflammation (*p* = 0.005). Moreover, VEGFR-2 staining intensity correlated with the expression level of ICAM-1 (*p* = 0.026). The expression of VEGFR-2, one of the main targets of antiangiogenic therapy, depends on GBM microenvironment. Higher endothelial VEGFR-2 levels were seen in the presence of more pronounced inflammation. Target dependence on inflammatory tumor microenvironment has to be taken into consideration when treatment approaches that block VEGFR-2 signaling are designed.

## 1. Introduction

Although considered as rare tumor entity, about 27700 new primary CNS cancers are diagnosed each year in Europe [1]. Out of these, glioblastoma multiforme (GBM) is the most aggressive and lethal type of a brain tumor in adults that accounts for approximately 20% of all malignant primary CNS tumors and 82% of high grade (WHO grades III and IV) gliomas [2, 3]. The prognosis of GBM patients is extremely dismal since current standard treatment options result only in medial survival times of 14.6 months [4].

GBM is one of the most angiogenic tumors. Therefore, in recent years, the inhibition of tumor angiogenesis has been an extremely attractive and dominating experimental therapeutic strategy in neurooncology [5, 6]. A number of anticancer drugs that block formation of new blood vessels through different molecular targets and patterns of action are currently in various stages of clinical development for both newly diagnosed and recurrent GBM [7]. However, first promising results of angiogenesis inhibitors, such as high radiological response rates and apparent clinical benefit, have been replaced by prevalent disappointment. Major limitations of antiangiogenic drugs used in GBM include the modest number of durable responses and the lack of cytotoxic antitumor activity and overall survival benefit [6]. The reasons of the lack of significant clinical efficacy of antiangiogenic drugs in GBM, however, are not fully elucidated [8].

In GBM, at least five mechanisms by which tumors achieve neovascularization have been described: vascular cooption, angiogenesis, vasculogenesis, vascular mimicry, and glioblastoma-endothelial cell transdifferentiation [9]. Out of these, angiogenesis and vasculogenesis have been most extensively studied and described. During angiogenesis, blood vessels arise from sprouting and proliferation of endothelial cells from preexisting vascular network, whereas in vasculogenesis,* de novo* blood vessels are formed through colonization of circulating bone-marrow-derived endothelial progenitor cells that are recruited to the tumor [9]. Both previously mentioned mechanisms of neovascularization are largely regulated via vascular endothelial growth factor (VEGF) and its receptor 2 (VEGFR-2) [10]. It has been shown that tumor microenvironment influences glioblastoma treatment outcome [11, 12]. The aim of the current study was to evaluate the impact of tumor microenvironment, particularly inflammatory reaction, on the expression of VEGFR-2, one of the main targets of antiangiogenic drugs.

## 2. Materials and Methods

Study was approved by the Research Ethics Committee of the University of Tartu, Estonia.

Surgically excised primary GBM tissues (*n* = 42) were obtained after primary operation (prior to radiotherapy) from patients treated at Tartu University Hospital or North Estonian Medical Centre.

### 2.1. Histology

Haematoxylin and eosin stained sections (4 *μ*m thick) were used for primary diagnosis. The diagnosis of GBM was confirmed by 2 independent pathologists. Afterwards, the overall extent of inflammatory reaction was estimated in tumor tissue by experienced pathologist. This was based on typical visual appearance of inflammation, including presence of edema and inflammatory cell infiltration. For the evaluation, an arbitrary score ranging from 1 to 3 was applied (1 = weak, 2 = moderate, and 3 = strong inflammatory reaction).

### 2.2. Immunohistochemistry (IHC)

GBM tissues were immunohistochemically examined for ICAM-1 and VEGFR-2 expression. Sections were cut from archived paraffin blocks and stained according to standard immunohistochemistry protocol. For immunohistochemistry, primary antibodies against ICAM-1 (Santa Cruz Biotechnology, Inc., #sc-8439, dilution 1 : 100) and VEGFR-2 (Santa Cruz Biotechnology, Inc., #sc-6251, dilution 1 : 100) were applied. Diaminobenzidine was used as chromogen.

The evaluation of immunohistochemically stained slides was carried out in a blinded fashion. First, immunohistochemical expression of intercellular adhesion molecule 1 (ICAM-1) was assessed. For this, digital IHC image analysis was performed. IHC digital image analysis was carried out in 6 selected images from each slide by using the freeware program ImageJ. The brown-colored area, occupied by the immunohistochemical reaction, was selected by the color threshold filtering tool to subtract the hematoxylin-stained areas at the background. Then the images were converted to the greyscale and the optical density by the area method was measured in pixel values ranging from 0 to 255. Value 0 represents the lightest shade of the color while 255 represents the darkest shade of the color in the image. Tissue expression of ICAM-1 was determined at a magnification of ×10.

The evaluation and scoring of VEGFR-2 immunohistochemically stained slides were carried out by 2 independent researchers. For VEGFR-2 expression, two parameters were assessed. First, the number of VEGFR-2 positive (VEGFR-2+) blood vessels per microscopic field was determined. Additionally, endothelial VEGFR-2 staining intensity was evaluated using an arbitrary score ranging from 0 to 3 (0 = no staining; 1 = weak, 2 = moderate, and 3 = strong staining intensity). For individual values, both parameters were determined in 5 microscopic fields. These values were used to evaluate the correlation between the assessments of 2 independent researchers. Afterwards, the mean number of VEGFR-2+ blood vessels and VEGFR-2 staining intensity in 10 microscopic fields (2 × 5 fields) were calculated. All VEGFR-2 parameters were determined in areas with vital tumor tissue (excluding necrotic areas) at a magnification of ×40.

### 2.3. Statistical Analysis

The SPSS statistical software was used to calculate individual means, group mean, and standard deviation of the mean. Additionally, Pearson correlation analysis was utilized. A *p* value <0.05 was regarded statistically significant.

## 3. Results

### 3.1. Histology and IHC

**Figure 1:**
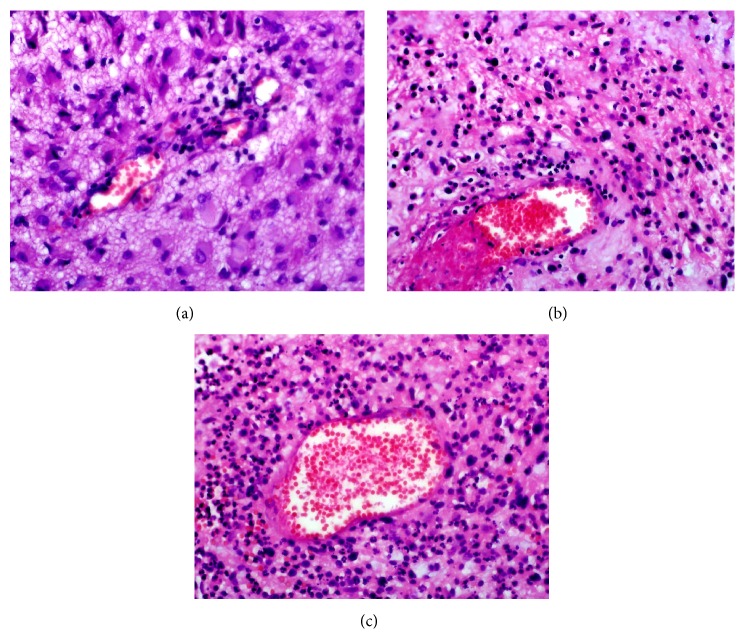
Inflammatory reaction in GBM. Photos illustrate GBM tissues with inflammatory reaction (photos represent different patients). (a) Weak (score 1) inflammation, (b) moderate (score 2) inflammation, and (c) strong (score 3) inflammation. Note the different numbers of tumor infiltrating inflammatory cells. Magnification ×40.

In individual GBM samples, the extent of inflammation varied, ranging from 1.0 to 3.0 and being in the whole group 1.9 ± 0.7 (mean ± SD).  Figure 1 represents GBM tissues with weak (a), moderate (b), and strong (c) visual inflammatory reaction.

**Figure 2:**
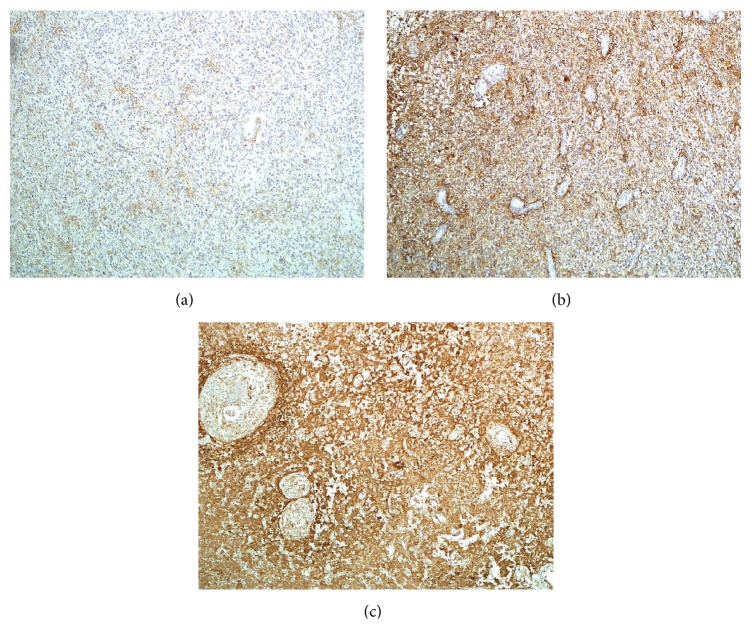
ICAM-1 expression in GBM. Photos illustrate GBM tissues with different extent of ICAM-1 expression (photos represent different patients). (a) Weak optical density, (b) moderate optical density, and (c) strong optical density. Magnification ×10.

Similarly, individual optical densities of ICAM-1 in GBM tissue varied, ranging from to 17.6 to 154.9 pixel values. Group mean optical density of ICAM-1 was 57.0 ± 27.1 (mean ± SD).  Figure 2 illustrates GBM tissues with weak (a), moderate (b), and strong (c) optical density of ICAM-1.

**Figure 3:**
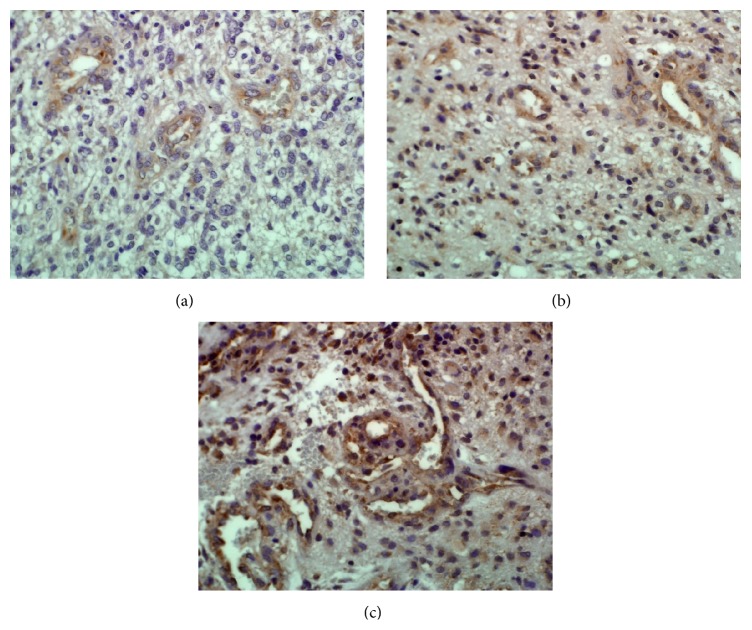
VEGFR-2 expression in GBM blood vessels. Photos illustrate GBM tissues with different endothelial staining intensities of VEGFR-2 in blood vessels (photos represent different patients). (a) Weak (score 1) staining intensity, (b) moderate (score 2) staining intensity, and (c) strong (score 3) staining intensity. Note also the different numbers of VEGFR-2+ blood vessels. Magnification ×40.

VEGFR-2 parameters were determined by 2 independent researchers whose results were in good accordance (*R* = 0.8, *p* < 0.0001). In GBM tissue sections, the number of VEGFR-2+ blood vessels per microscopic field and endothelial VEGFR-2 staining intensity were 6.2 ± 2.4 (mean ± SD; range 2.8–13.5) and 1.2 ± 0.8 (mean ± SD; range 0.0–2.8), respectively.  Figure 3 illustrates GBM tissues with weak (a), moderate (b), and strong (c) expression level of VEGFR-2 in tumor blood vessels.

### 3.2. Correlation Analysis

The results of correlation analysis are described in Table 1. A positive association was found between the extent of visual inflammation and endothelial VEGFR-2 staining intensity (*p* = 0.005). Moreover, endothelial VEGFR-2 staining intensity correlated with the expression level of tissue ICAM-1 (*p* = 0.026). Additionally, there was a trend toward significant association between the number of VEGFR-2 positive blood vessels and endothelial VEGFR-2 staining intensity in GBM tissue (*p* = 0.065).

## 4. Discussion

Downstream effects of VEGFR-2 activation in the vascular endothelium include cell proliferation, migration, permeability, and survival, resulting in neovascularization processes, such as angiogenesis and vasculogenesis [10]. Consequently, this receptor has been very attractive target in the development of antiangiogenic drugs (e.g., bevacizumab, sunitinib, sorafenib, vatalanib, vandetanib, recentin, and cediranib) [10]. Unfortunately, a number of these antiangiogenic drugs (vandetanib, cediranib, sorafenib, and sunitinib) have failed to show clinical efficacy in different phases of clinical trials in both newly diagnosed and recurrent glioblastoma [13–17]. Moreover, the most advanced antiangiogenic drug in glioblastoma, bevacizumab, did not get approval from The European Medicines Agency Committee for Medicinal Products for Human Use (CHMP) due to the lack of clinically relevant efficacy [18, 19]. All these negative trials have caused a lot of frustration since the results do not coincide with the initial expectations. The reasons of the lack of significant clinical efficacy of antiangiogenic drugs, however, are not fully elucidated.

In the present study, we evaluated the impact of tumor microenvironment on the expression level of VEGFR-2, one of the main targets of antiangiogenic drugs. Foremost, the possible role of inflammatory reaction was assessed. Inflammatory reaction was evaluated by two means. First, visual inflammation (based on the presence of tissue edema and inflammatory cell infiltration) was estimated in hematoxylin-eosin stained sections by experienced pathologist. Afterwards, to reduce subjectivity, a digital IHC image analysis was performed in ICAM-1 stained sections. ICAM-1 was chosen as a marker of inflammation since this transmembrane glycoprotein can be induced in response to a number of stimuli, including inflammatory mediators, hormones, and cellular stresses [20, 21]. Moreover, endothelial ICAM-1 is considered to represent the most important adhesion molecule for leukocyte recruitment to inflamed sites [22–24].

All glioblastoma samples showed various levels of visually confirmed inflammatory reaction. This is not surprising since inflammation is considered one of the characteristic histopathological features of glioblastoma [25]. Also, the expression of ICAM-1 was present in all digitally analyzed individual tumor samples, which is in good accordance with previous studies, where compared to peritumoral ICAM-1 expression significantly higher expression of ICAM-1 has been detected in GBM tumor areas in both gene and protein levels [26–28]. In GBM cells, ICAM-1 expression has been shown to increase following stimulation with proinflammatory cytokines, such as interleukin-1*β* (IL-1*β*), tumor necrosis factor-alpha (TNF*α*), and interferon-gamma (IFN-*γ*) [26, 29], indicating that ICAM-1 is one of the inflammatory mediators also in this type of cancer.

In GBM tissues, different numbers of VEGFR-2+ blood vessels and endothelial levels of VEGFR-2 were detected. Previous studies have shown that in normal brain, low or undetectable endothelial expression of VEGFR-2 can be found; however, in gliomas, the proportion of VEGFR-2+ vessels and endothelial VEGFR-2 expression increases with tumor grade, being the highest in GBM [30, 31]. Our study revealed that also in the most aggressive glioma—GBM—the extent of VEGFR-2 expression may vary. Additionally, present study showed that the expression of VEGFR-2 depends on inflammatory reaction in tumor tissue: the higher the endothelial VEGFR-2 expression the higher the extent of inflammation. Moreover, this association was seen for both assessments of inflammatory reaction (visual and computer software based).

Angiogenesis is a tightly controlled process that in a number of pathological conditions, including cancer and inflammation, may become aberrant [32]. Different factors produced by tissues are capable of promoting or inhibiting blood vessel proliferation, whereas in normal status, the balance between angiogenic and angiostatic factors exists. In inflammation, this balance is clearly inclined toward angiogenic factors and angiogenesis [33].

Although the link between inflammation and angiogenesis has received much attention only recently, there is a substantial body of evidence showing close association between these two processes. Previous studies have described that angiogenic factors exhibit both proangiogenic and proinflammatory effects, inflammatory cells are able to produce large quantities of proangiogenic factors, and both processes (inflammation and angiogenesis) are capable of potentiating each other [33]. For example, VEGF that exerts majority of its angiogenic effects by binding to VEGFR-2 has also been shown to induce adhesion molecules on endothelial cells during inflammation [34]. In endothelial cells, treatment with VEGF results in an increase of both ICAM-1 mRNA and protein expression [35]. Moreover, VEGF increases leukocyte adhesiveness to endothelial cells, which is the first step of leukocyte trafficking into inflamed tissue [35]. Next to these effects, VEGF enhances vascular permeability and causes vasodilatation, potentiating thereby inflammation through formation of tissue edema [10, 36]. At the same time, hyperpermeability is also involved in pathological angiogenesis [36]. Additionally, inflammatory and angiogenic processes involve similar cell types. Inflammatory cells, namely, monocytes, macrophages, T lymphocytes, and neutrophils, participate in the angiogenesis by secreting cytokines that affect endothelial cell functions, proliferation, migration, and activation [37]. Macrophages, present in the inflammatory infiltrate, produce a broad array of angiogenic growth factors and cytokines, generate channels for blood flow through proteolytic mechanisms, and promote the remodeling of arterioles into arteries [32]. Inflammatory dentritic cells stimulate similarly angiogenesis by secreting angiogenic factors and cytokines, as well as by promoting proangiogenic activity of T lymphocytes [32]. Previous studies have also shown that proinflammatory cytokines, which are always present in inflamed tissue, mediate also endothelial expression of VEGFR-2 [38, 39]. Later this is also indirectly supported by our findings since positive correlation was found between the extent of VEGFR-2 expression and inflammatory response in GBM tissue.

There are several clinical situations, where inflammatory reaction in GBM may be suppressed. These particularly include the use of anti-inflammatory drugs, such as steroids and nonsteroidal anti-inflammatory drugs (NSAIDs), to manage tumor surrounding inflammation and edema [40]. Whether these very commonly used medicines influence also treatment efficacy of antiangiogenic drugs through diminishing inflammatory response and thereby the expression of VEGFR-2 remains unclear. Nevertheless, our data point toward the possibility that this association might exist. This is also supported by studies where dexamethasone, most frequently used steroid in GBM patients, has been shown to inhibit the effects of proinflammatory cytokines, VEGF mRNA expression, VEGFR-2 expression, and macrophage infiltration [39, 41, 42].

## 5. Conclusions

In conclusion, our study showed that the expression of VEGFR-2, one of the main targets of antiangiogenic drugs, depends on GBM microenvironment. Importantly, higher endothelial VEGFR-2 levels were seen in the presence of more pronounced inflammation, whereas in less inflamed tissues only weak expression of VEGFR-2 was found. Target dependence on inflammatory tumor microenvironment has to be taken into consideration when treatment approaches that block VEGFR-2 signaling are designed.

## Figures and Tables

**Table 1:** Results of correlation analysis^*∗*^.

Correlations	*p* value
Visual inflammatory reaction and VEGFR-2 staining intensity	*p* = 0.005
Tissue ICAM-1 expression and VEGFR-2 staining intensity	*p* = 0.026
The number of VEGFR-2+ blood vessels and VEGFR-2 staining intensity	*p* = 0.065

^*∗*^Bivariate Pearson correlation test.
